# Supplementary material for: Exposure to SARS-CoV-2 generates T-cell memory in the absence of a detectable viral infection
Source: Nat Commun. 2021 Mar 19;12:1724. doi: 10.1038/s41467-021-22036-z (PMC7979809; doi:10.1038/s41467-021-22036-z)
Supplement: Supplementary file 1 — Supplementary Information [file 41467_2021_22036_MOESM1_ESM.pdf]

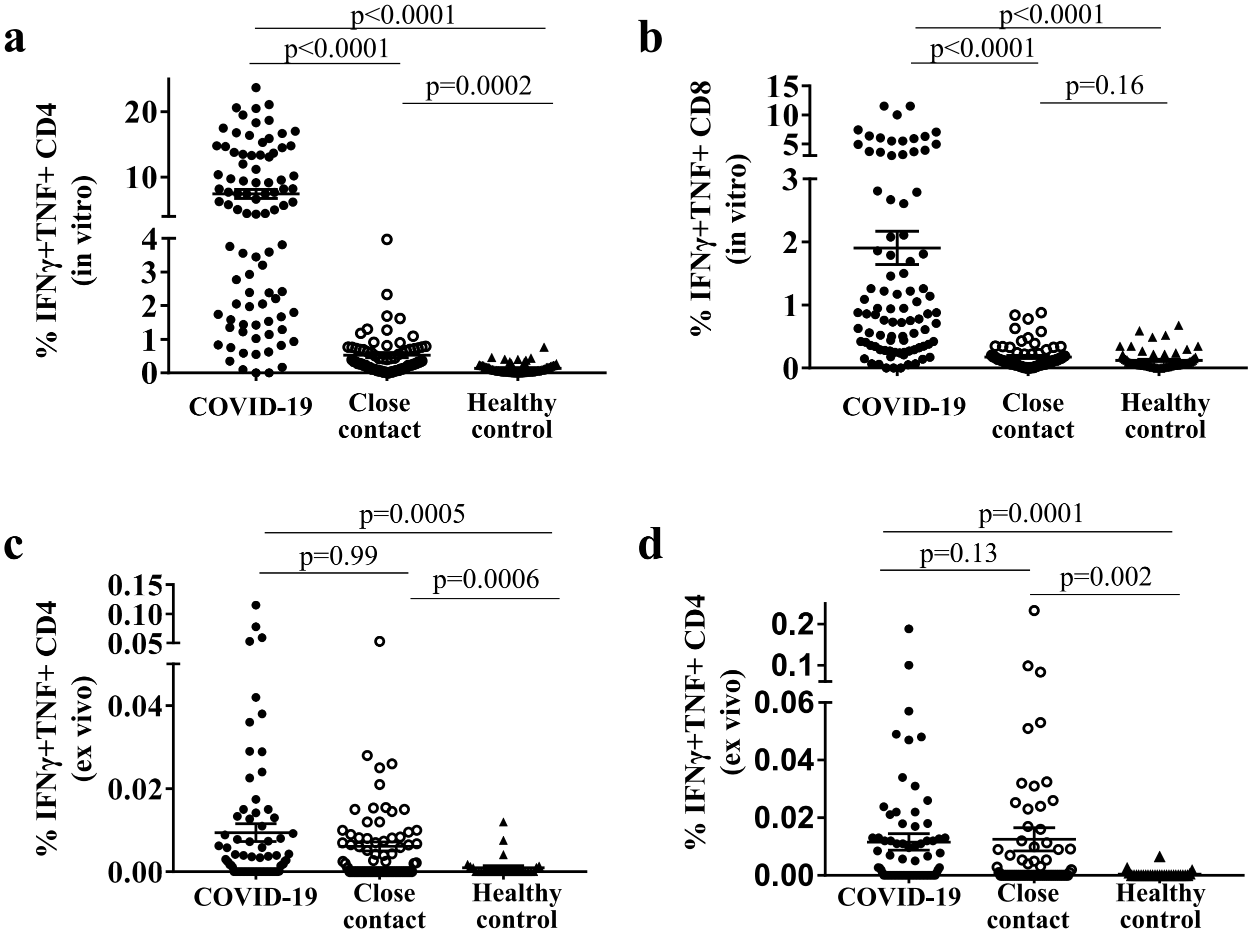

Supplementary Figure 1: Dual expression of IFNγ and TNF in SARS-CoV-2 peptide stimulated T cells in recovered COVID-19 patients, close contacts and unexposed healthy donors. Graphs show the frequency of IFNγ and TNF dual expressing cells in $CD4^+$ (a) and $CD8^+$ (c) T cells in PBMCs from 10-day culture after stimulation_(n=90 for COVID-19, n=69 for close contacts and n=63 for healthy donors), and in $CD4^+$ (b) and $CD8^+$ (d) T cells in PBMCs stimulated overnight_(n=89 for COVID-19, n=69 for close contacts and n=30 for healthy donors). Data are presented as mean frequencies of $IFN\gamma^+CD4^+/CD8^+$ T cells +/- SEM. Student's t test was performed with two-sided p value as indicated.

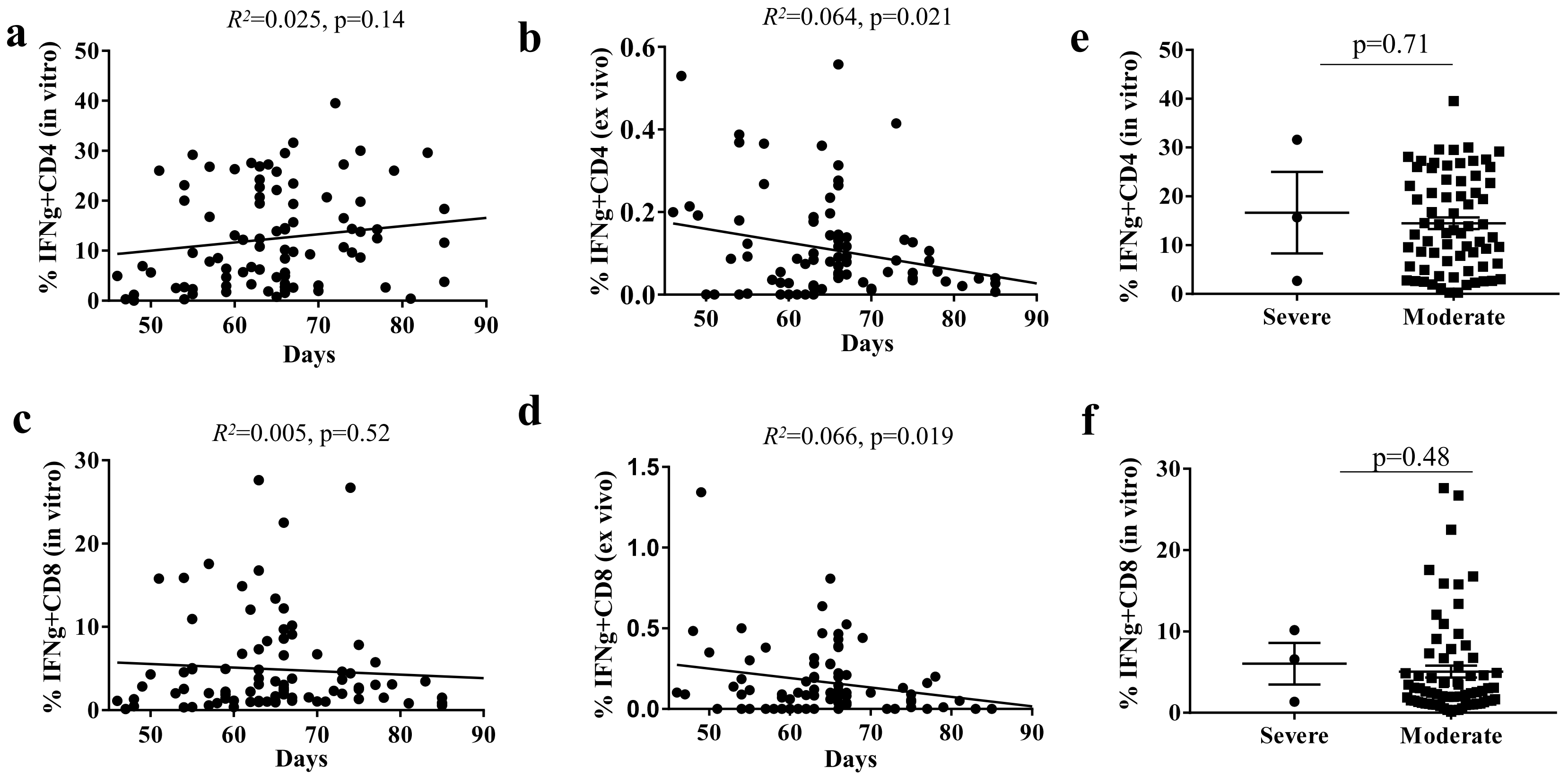

Supplementary Figure 2: No change in IFNγ producing $CD4^+$ or $CD8^+$ T cells over time. PBMCs were stimulated overnight or expanded for 10 days with SARS-CoV-2 peptides and immuno-stained. IFNγ expressing cells were analyzed with FACS. Correlations of in vitro expansion (a, c) (n=90) and overnight stimulation (b, d) (n=89) of the $IFN\gamma^+$ $CD4^+$ (a, b) and $CD8^+$ (c, d) T cells with the days upon disease onset. (e, f) Comparison of in vitro $CD4^+$ (e) and $CD8^+$ (f) T cell expansion between patients after recovery from severe (n=3) or moderate disease (n=69). Statistical analysis was carried out with the linear regression method, with *p* values as indicated for a-d. Student's *t test* was performed with two-sided *p* values as indicated for e and f. Data are presented as mean frequencies of $IFN\gamma^+CD4^+/CD8^+$ T cells +/- SEM.

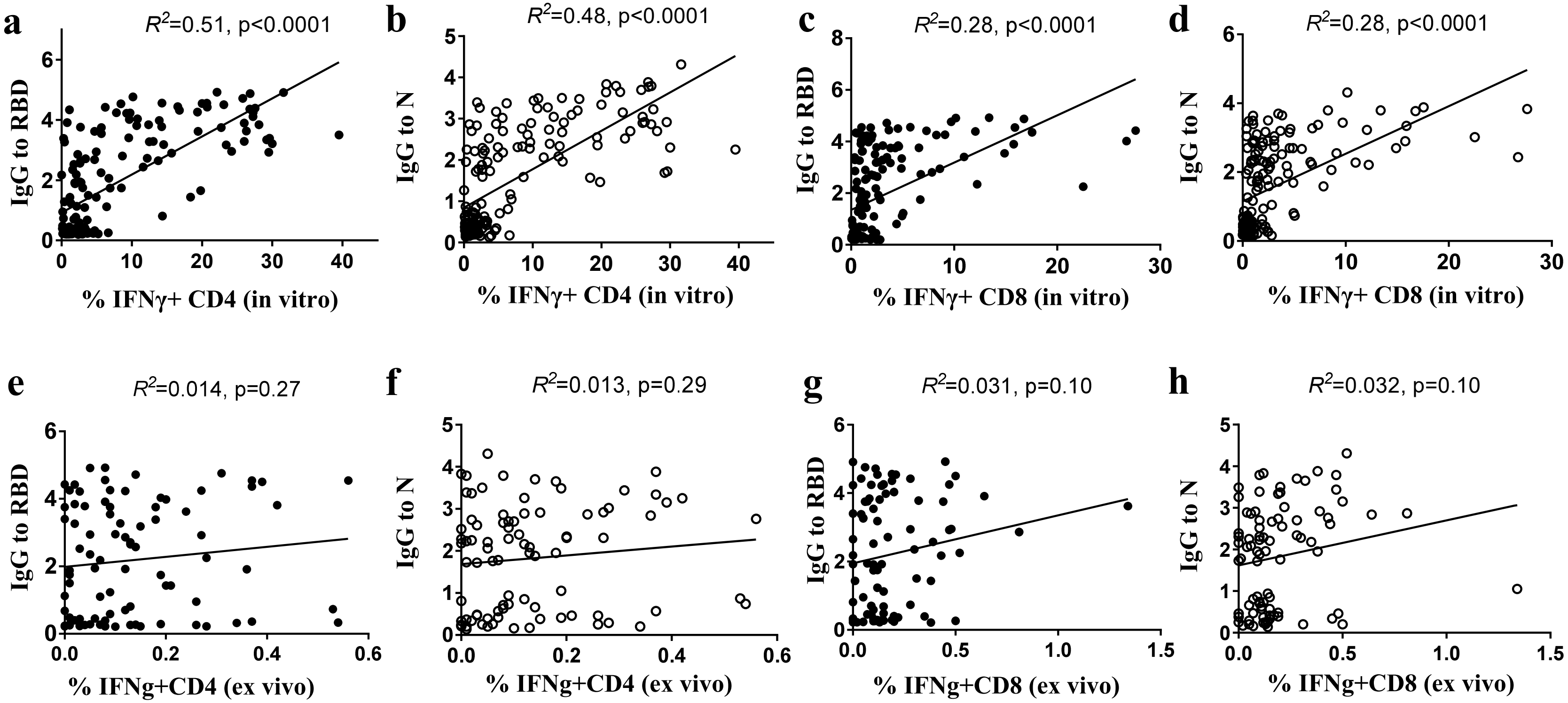

Supplementary Figure 3: Correlations between the T cell immunity and antibody responses against SARS-CoV-2. PBMCs were stimulated for overnight or expanded for 10 days after stimulation with SARS-CoV-2 peptides and immunostained as described in Figure 1 and the Methods section. Antibodies presence in the blood plasma were determined with ELISA. Top panel: Frequencies of IFNγ expressing $CD4^+$ (a, b) and $CD8^+$ (c, d) T cells are plotted against the antibody titers specific to SARS-CoV-2 RBD (a and c, closed circle) and N protein (c and d, open circle) after 10-day expansion (n=159). Bottom panel: Frequencies of IFNγ expressing $CD4^+$ (e, f) and $CD8^+$ (g, h) T cells are plotted against the antibody titers specific to SARS-CoV-2 RBD (e and g, closed circle) and N protein (f and h, open circle) after overnight stimulation (n=89). Statistical analysis was carried out with linear regression method, with two-sided p values as indicated. Data were pooled from multiple experiments.

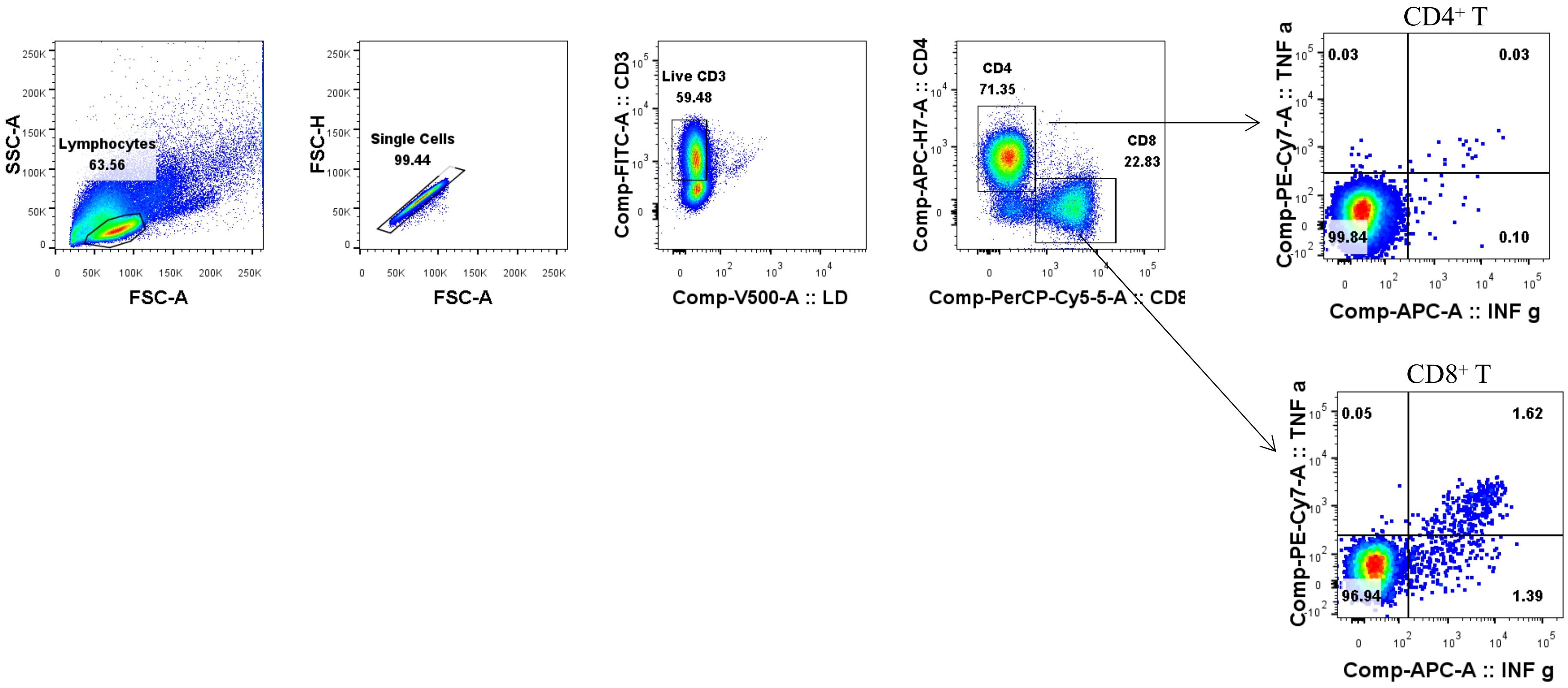

Supplementary Figure 4: Gating strategy for our flow cytometry experiments. PBMCs were stimulated for overnight or expanded for 10 days after stimulation with SARS-CoV-2 peptides and then were immuno-stained for surface and intracellular. Live CD3 were gated from singlets derived from lymphocytes, based on live CD3, CD4 and CD8 were further gated on IFNγ and TNF. The gating strategy was applied to generation of data at Figure1-3 and supplementary Figure 1-3.

Supplementary Table 1. Demographics and antibody titer of all subjects.

Subject No.	Symptoms	Age	Gender	days from disease onse	IgG（N+S）	IgG (N)	IgG（S RBD）	IgM （N+S）	nAb unit
co-015	A	59	F	48	41.6	1.3	2.2	4.0	309
co-038	A	16	M	66	152.2	2.1	2.9	1.0	161
co-051	A	13	F	57	97.6	2.2	2.4	3.5	287
co-086	A	32	M	67	91.7	2.9	3.9	25.9	655
co-089	A	71	F	49	21.9	1.1	1.7	0.9	136
co-090	A	56	F	67	131.3	2.9	3.6	3.3	1984
co-092	A	13	M	48	95.4	1.8	2.2	1.1	191
co-096	A	64	F	55	144.3	2.7	3.5	4.0	1038
co-097	A	66	M	55	27.3	0.7	1.2	0.7	236
co-102	A	51	F	66	77.7	2.1	2.7	5.5	289
co-107	A	31	F	83	5.0	0.5	0.4	0.5	
co-108	A	52	M	83	16.5	0.8	1.1	0.6	
co-109	A	52	F	83	30.5	1.7	2.5	6.1	
co-110	A	55	F	74	84.1	2.7	4.3	3.4	
co-137	A	16	F	85	72.3	1.7	1.5	5.9	
co-144	A	36	F	78	107.8	2.0	2.5	4.1	
co-155	A	11	F	77	101.5	2.3	3.3	11.0	
co-159	A	39	F	64	6.3	0.4	1.1	1.1	
		**Mean**	**%M**	**Mean**	**Mean**	**Mean**	**Mean**	**Mean**	**Mean**
		**41**	**28**	**67**	**72.5**	**1.8**	**2.4**	**4.6**	**569**
co-001	M	39	F	64	118.9	2.8	1.9	7.9	190
co-002	M	53	M	66	71.2	3.0	2.3	6.7	1161
co-003	M	50	F	66	103.9	2.9	2.9	7.0	1209
co-005	M	60	M	63	107.8	3.8	4.9	4.5	294
co-006	M	52	M	63	145.8	3.4	4.4	3.3	726
co-007	M	52	F	65	102.6	2.3	4.0	1.0	1739
co-008	M	45	M	63	114.1	2.4	3.6	5.7	1045
co-009	M	40	M	60	116.7	3.0	3.3	6.5	635
co-011	M	33	F	62	143.8	3.2	4.4	6.6	862
co-012	M	62	F	57	139.9	3.9	4.4	29.7	839
co-013	M	36	F	55	77.4	1.7	3.3	3.3	340
co-014	M	36	M	54	65.5	1.6	2.6	1.1	697
co-017	M	52	F	54	116.0	3.2	4.5	26.9	935
co-018	M	62	F	54	96.7	3.3	4.5	4.6	1175
co-022	M	65	F	51	100.6	2.9	3.9	5.6	1014
co-023	M	53	F	57	131.6	3.5	4.3	50.2	925
co-024	M	62	F	60	134.1	2.9	3.6	17.4	746
co-027	M	57	M	48	15.6	0.5	1.4	0.5	394
co-029	M	46	M	66	98.0	2.8	4.5	31.0	899
co-030	M	43	F	64	135.6	3.8	4.3	31.5	235
co-032	M	53	M	61	151.2	3.3	2.7	1.2	527
co-035	M	56	M	63	112.8	3.8	4.4	38.6	1227
co-036	M	71	M	50	76.9	2.2	3.8	2.1	415
co-037	M	48	M	66	86.7	2.2	1.9	0.9	311
co-039	M	70	F	66	129.6	3.4	4.8	1.1	1841
co-042	M	25	M	63	142.0	3.5	4.0	4.1	511
co-043	M	47	F	73	143.4	2.9	4.1	3.0	1176
co-045	M	40	M	66	133.2	2.9	3.2	19.4	546
co-046	M	45	F	66	156.7	3.5	3.8	3.3	360
co-049	M	34	F	55	161.4	3.3	2.9	7.7	1447
co-050	M	42	M	53	18.9	0.9	0.6	5.6	47
co-053	M	56	M	57	108.1	2.3	4.2	2.7	602
co-054	M	57	F	55	84.7	2.3	3.4	6.7	260
co-057	M	42	M	58	109.7	2.3	1.7	9.7	211
co-061	M	30	M	62	40.1	1.2	2.1	1.5	152
co-062	M	31	M	63	74.8	2.7	3.8	3.8	694
co-064	M	35	F	47	20.6	0.9	0.7	0.6	89
co-065	M	37	M	54	88.1	2.0	3.4	3.7	167
co-066	M	40	F	67	86.4	1.9	2.6	1.1	573
co-067	M	51	F	71	166.8	3.6	4.6	4.9	380

co-068	M	51	M	67	110.4	2.9	4.2	4.3	999
co-072	M	37	F	63	162.0	3.7	3.8	38.6	437
co-077	M	27	F	46	88.8	2.3	1.4	3.5	1105
co-093	M	32	F	63	131.6	2.7	3.0	13.4	545
co-100	M	16	M	75	70.5	1.6	2.8	5.9	452
co-101	M	19	F	66	69.0	2.0	2.7	3.5	358
co-104	M	50	M	59	142.4	3.2	2.7	5.5	290
co-112	M	54	M	74	126.6	3.2	4.4	7.3	
co-113	M	52	F	75	108.0	2.4	4.0	2.7	
co-114	M	52	M	71	91.8	2.2	1.7	11.0	
co-115	M	53	M	70	87.3	2.6	4.2	26.8	
co-118	M	57	M	66	156.7	3.8	4.9	46.8	
co-119	M	77	F	66	155.9	3.7	4.7	120.6	
co-120	M	53	M	68	135.1	2.5	4.3	1.4	
co-121	M	52	F	58	126.5	2.5	3.2	26.6	
co-122	M	41	M	75	115.5	2.3	3.2	8.3	
co-126	M	48	M	77	102.4	2.4	3.3	12.0	
co-128	M	46	F	79	115.1	2.7	3.8	6.8	
co-133	M	37	M	79	151.8	3.0	3.3	2.8	
co-134	M	42	M	83	72.2	1.7	3.4	2.4	
co-135	M	41	F	86	85.4	1.6	1.4	5.1	
co-136	M	19	M	85	94.4	2.1	2.4	14.3	
co-138	M	32	M	75	70.1	1.5	1.7	4.9	
co-148	M	42	F	73	119.5	3.3	3.8	17.3	
co-152	M	39	M	77	126.8	2.7	4.6	13.6	
co-153	M	60	F	72	105.9	2.3	3.5	6.6	
co-156	M	51	M	70	127.2	3.4	1.9	6.7	
co-157	M	50	F	67	73.1	2.5	0.7	3.5	
co-158	M	20	M	65	98.5	1.8	1.9	1.9	
co-069	S	62	M	67	50.1	3.1	2.9	116.3	1997
co-085	S	59	M	67	135.5	4.3	4.9	15.1	523
co-099	S	49	M	66	81.8	2.3	3.8	8.9	689
		**Mean**	**%M**	**Mean**	**Mean**	**Mean**	**Mean**	**Mean**	**Mean**
		**46**	**55.5**	**65**	**107.2**	**2.7**	**3.3**	**13.0**	**700**
co-004	H	37	M		0.8	0.4	0.2	0.6	
co-010	H	64	F		0.3	0.3	0.8	4.0	
co-016	H	61	M		0.9	0.3	0.3	5.4	
co-019	H	61	M		1.6	0.3	0.4	0.5	
co-020	H	66	M		0.3	0.3	0.2	0.6	
co-021	H	39	M		3.1	0.3	0.3	0.5	
co-025	H	63	M		1.7	0.7	0.3	0.3	
co-026	H	36	M		0.9	0.5	0.2	0.3	
co-028	H	24	M		0.3	0.2	0.3	0.4	
co-031	H	45	M		0.4	0.7	0.3	0.8	
co-033	H	56	F		1.7	0.6	0.2	1.1	
co-034	H	35	M		0.6	0.3	0.2	0.6	
co-040	H	47	F		0.9	0.4	0.3	15.8	
co-041	H	51	M		0.7	0.3	0.2	1.3	
co-044	H	47	M		1.5	0.3	0.3	2.1	
co-047	H	70	F		0.2	0.6	0.4	2.7	
co-048	H	45	M		0.2	0.6	0.3	0.6	
co-052	H	12	F		0.6	0.3	0.3	0.8	
co-055	H	18	M		0.3	0.2	0.2	2.3	
co-056	H	23	M		0.9	0.6	0.4	1.0	
co-058	H	18	F		0.5	0.5	1.0	0.8	
co-059	H	38	F		1.7	0.5	0.3	0.3	
co-060	H	17	F		0.3	0.3	0.3	0.8	
co-063	H	31	F		0.5	0.5	0.2	0.8	
co-070	H	24	M		0.2	0.2	0.2	0.4	
co-071	H	37	M		0.3	0.5	0.3	0.3	
co-073	H	8	M		1.0	0.2	0.3	0.4	
co-074	H	42	M		34.9	0.9	0.7	1.6	
co-075	H	53	M		0.8	0.7	0.3	1.2	
co-076	H	71	M		0.4	0.6	0.4	0.6	
co-078	H	58	M		1.5	0.2	0.3	0.5	
co-079	H	12	M		0.8	0.3	0.2	0.5	
co-080	H	12	F		0.8	0.3	0.2	0.5	

co-081	H	40	F	0.2	0.2	0.2	1.0
co-082	H	32	F	0.5	0.7	0.2	1.0
co-083	H	10	F	0.5	0.3	0.3	0.8
co-084	H	56	F	0.2	0.2	0.2	0.8
co-087	H	47	M	0.2	0.1	0.3	0.5
co-088	H	50	M	0.3	0.2	0.3	1.1
co-091	H	34	F	0.2	0.2	0.2	1.3
co-094	H	34	M	0.1	0.2	0.2	0.9
co-095	H	7	F	0.1	0.2	1.1	0.4
co-098	H	35	M	0.4	0.3	0.2	0.5
co-103	H	48	M	0.1	0.5	1.3	1.2
co-105	H	49	F	0.3	0.3	0.2	0.6
co-106	H	48	F	0.3	0.6	0.2	0.5
co-111	H	42	M	0.5	0.2	0.2	0.3
co-116	H	12	F	0.7	0.4	0.4	0.7
co-117	H	50	F	0.3	0.3	0.4	0.8
co-123	H	20	M	0.2	0.3	0.2	0.6
co-124	H	12	F	1.4	0.2	0.3	3.5
co-125	H	43	F	0.3	0.5	0.3	0.5
co-127	H	70	M	1.1	0.6	0.3	0.4
co-129	H	22	F	0.3	0.4	0.5	1.5
co-130	H	52	M	1.3	0.5	0.5	0.6
co-131	H	35	F	0.5	0.5	0.4	1.2
co-132	H	58	F	0.3	0.4	0.2	0.5
co-139	H	58	M	0.2	0.2	0.3	1.1
co-140	H	31	F	0.2	0.2	0.3	2.2
co-141	H	55	F	0.5	0.5	0.2	0.5
co-142	H	54	F	0.2	0.2	0.3	0.8
co-143	H	9	M	0.4	0.2	0.3	0.6
co-145	H	41	M	0.2	0.2	0.2	2.3
co-146	H	14	M	0.2	0.1	0.2	0.5
co-147	H	75	M	1.4	0.4	0.2	0.3
co-149	H	7	M	6.6	0.3	0.3	0.7
co-150	H	60	M	0.3	0.2	0.3	0.4
co-151	H	32	M	0.4	0.4	0.3	0.3
co-154	H	36	F	1.3	0.2	0.3	1.1
		**Mean**	**%M**	**Mean**	**Mean**	**Mean**	**Mean**
		**39**	**57.9**	**1.2**	**0.2**	**0.3**	**1.2**

A; asymptomatic: M: mild-moderate; S: severe, H; healthy (NAT-Ab-). nAb: neutralizing antibody titer. NA: not applicable; ND: not detectable.

Supplementary Table 2.Validation of three Ab assays

	Assay 1 immunochromatographic		Assay 2 immunochromatographic		Assay 3 ELISA	
	IgG N+S	IgM* N+S	IgG N+S	IgM* N+S	IgG N	IgG sRBD
Number of negative controls for method validation	284	286	188	188	403	269
Minimum	0.00	0.00	0.29	0.17	0.11	0.09
25% Percentile	0.08	0.14	1.23	0.81	0.24	0.18
Median	0.18	0.24	1.87	1.46	0.35	0.21
75% Percentile	0.43	0.38	3.20	3.01	0.47	0.26
Maximum	1.79	1.91	18.73	11.60	0.97	0.62
**Mean**	**0.26**	**0.30**	**2.67**	**2.19**	**0.38**	**0.23**
Std. Deviation	0.23	0.26	2.49	2.03	0.19	0.09
Std. Error of Mean	0.01	0.02	0.18	0.15	0.01	0.01
Establishment of statistic parameters used this study with the 79 confirmed covid-19 patients						
Theoretical cut off (Mean+3SD)	0.95	1.08	10.15	8.27	0.95	0.55
**Cut off used in test**	**1**	**1.1**	**11**	**10**	**1**	**1**
False positive (%)	0.70	3.85	1.06	1.06	0	0
Specificity: Consistency with NAT result (% of 79 confirmed cases)	93.6	53.1¶	97.4	26.5¶	91.1	94.9

*: Reliable serum IgM titer is only detected within the first 3 weeks from disease onset (Fig. 3c)

¶: It was 48-86 days between their hospitalisation and the date of IgM assay for these 79 confirmed covid-19 patients.
