# Supplementary material for: Exposure to SARS-CoV-2 generates T-cell memory in the absence of a detectable viral infection
Source: Nat Commun. 2021 Mar 19;12:1724. doi: 10.1038/s41467-021-22036-z (PMC7979809; doi:10.1038/s41467-021-22036-z)
Supplement: Supplementary file 3 — Reporting Summary [file 41467_2021_22036_MOESM3_ESM.pdf]

nature research

Corresponding author(s): Pixin Ran

Last updated by author(s): Feb 1, 2020

Reporting Summary

Nature Research wishes to improve the reproducibility of the work that we publish. This form provides structure for consistency and transparency in reporting. For further information on Nature Research policies, see our Editorial Policies and the Editorial Policy Checklist.

Statistics

For all statistical analyses, confirm that the following items are present in the figure legend, table legend, main text, or Methods section.

n/a	Confirmed	
☐	☒	The exact sample size (n) for each experimental group/condition, given as a discrete number and unit of measurement
☐	☒	A statement on whether measurements were taken from distinct samples or whether the same sample was measured repeatedly
☐	☒	The statistical test(s) used AND whether they are one- or two-sided<br>*Only common tests should be described solely by name; describe more complex techniques in the Methods section.*
☐	☒	A description of all covariates tested
☐	☒	A description of any assumptions or corrections, such as tests of normality and adjustment for multiple comparisons
☐	☒	A full description of the statistical parameters including central tendency (e.g. means) or other basic estimates (e.g. regression coefficient) AND variation (e.g. standard deviation) or associated estimates of uncertainty (e.g. confidence intervals)
☐	☒	For null hypothesis testing, the test statistic (e.g. F, t, r) with confidence intervals, effect sizes, degrees of freedom and P value noted<br>*Give P values as exact values whenever suitable.*
☒	☐	For Bayesian analysis, information on the choice of priors and Markov chain Monte Carlo settings
☒	☐	For hierarchical and complex designs, identification of the appropriate level for tests and full reporting of outcomes
☐	☒	Estimates of effect sizes (e.g. Cohen's d, Pearson's r), indicating how they were calculated

Our web collection on statistics for biologists contains articles on many of the points above.

Software and code

Policy information about availability of computer code

Data collection	BD FACSDiva™ Software v8.0.1
Data analysis	FlowJo 10.6.2., GraphPad Prism 7.

For manuscripts utilizing custom algorithms or software that are central to the research but not yet described in published literature, software must be made available to editors and reviewers. We strongly encourage code deposition in a community repository (e.g. GitHub). See the Nature Research guidelines for submitting code & software for further information.

Data

Policy information about availability of data

All manuscripts must include a data availability statement. This statement should provide the following information, where applicable:

- Accession codes, unique identifiers, or web links for publicly available datasets
- A list of figures that have associated raw data
- A description of any restrictions on data availability

The data that support the findings of this study are available from the corresponding author upon reasonable request.

Field-specific reporting

Life sciences study design

All studies must disclose on these points even when the disclosure is negative.

Sample size	We take n>30 as a criteria for clinical samples examined.
Data exclusions	No data were excluded from the analyses.
Replication	Our study involves large sample sizes, including 222 samples for in vitro assays and among of the 222 samples, 188 were further analyzed with ex vivo assays. The in vitro assays were performed in four sets of experiments and the in vitro assays were performed in 8 sets of experiments. The percentage of the reactive CD4+ T cells and CD8+ T cells in the specific group did not vary significantly. These suggest a reproducibility of our findings.
Randomization	The 222 samples for in vitro assays and 188 samples for the ex vivo assays were randomly divided into 4 and 8 sets of experiments.
Blinding	Experiment designer allocate the ID number and the experiment performer/analyser did not know the ID for patients, close contacts and healthy donor, it is double blinded experiments.

Reporting for specific materials, systems and methods

We require information from authors about some types of materials, experimental systems and methods used in many studies. Here, indicate whether each material, system or method listed is relevant to your study. If you are not sure if a list item applies to your research, read the appropriate section before selecting a response.

Materials & experimental systems

n/a	Involved in the study	
☐	☒	Antibodies
☒	☐	Eukaryotic cell lines
☒	☐	Palaeontology and archaeology
☒	☐	Animals and other organisms
☐	☒	Human research participants
☒	☐	Clinical data
☒	☐	Dual use research of concern

Methods

n/a	Involved in the study	
☒	☐	ChIP-seq
☐	☒	Flow cytometry
☒	☐	MRI-based neuroimaging

Antibodies

Antibodies used	Antibody information is provided in details accordingly in the methods section of the manuscript.
Validation	All of the antibodies were purchased from BD biosciences, BD Pharmingen™ and BioLegend, which have clear data for antibody validation for the specific clone of antibodies.

Human research participants

Policy information about studies involving human research participants

Population characteristics	The information is provided accordingly in methods section and in supplementary Table 1.
Recruitment	Recruited by phone calls to all the COVID-19 recovered patients in two local hospitals and whoever agreed to join the cohort, together with their family close contacts.
Ethics oversight	Provided accordingly.

Note that full information on the approval of the study protocol must also be provided in the manuscript.

Flow Cytometry

Plots

Confirm that:

- [x] The axis labels state the marker and fluorochrome used (e.g. CD4-FITC).
- [x] The axis scales are clearly visible. Include numbers along axes only for bottom left plot of group (a 'group' is an analysis of identical markers).
- [x] All plots are contour plots with outliers or pseudocolor plots.
- [x] A numerical value for number of cells or percentage (with statistics) is provided.

Methodology

Sample preparation	PBMC isolation and antibody staining were clearly described in the methods section of the manuscript.
Instrument	BD FACSAria™ III.
Software	Data were acquired by BD FACSDiva™ Software v8.0.1, analyzed by FlowJo 10.6.2., and GraphPad Prism 7.
Cell population abundance	n/a
Gating strategy	Gating strategy is provided in the supplementary information file and cell populations are defined.

- [x] Tick this box to confirm that a figure exemplifying the gating strategy is provided in the Supplementary Information.
